# 3D bioprinting microgels to construct implantable vascular tissue

**DOI:** 10.1111/cpr.13456

**Published:** 2023-05-17

**Authors:** Xinhuan Wang, Xin Liu, Wenli Liu, Yanyan Liu, Ailing Li, Dong Qiu, Xiongfei Zheng, Qi Gu

**Affiliations:** ^1^ State Key Laboratory of Membrane Biology Institute of Zoology, Chinese Academy of Sciences Chaoyang District Beijing 100101 P. R. China; ^2^ School of Materials Design and Engineering, Beijing Institute of Fashion Technology Chaoyang District Beijing 100029 P. R. China; ^3^ Beijing National Laboratory for Molecular Sciences, State Key Laboratory of Polymer Physics and Chemistry Institute of Chemistry, Chinese Academy of Sciences Haidian District Beijing 100190 P. R. China; ^4^ University of Chinese Academy of Sciences Huairou District Beijing 101449 P. R. China; ^5^ Shenyang Institute of Automation, Chinese Academy of Sciences Hunnan District Shenyang 110169 P. R. China; ^6^ Beijing Institute for Stem Cell and Regenerative Medicine Chaoyang District Beijing 100101 P. R. China

## Abstract

Engineered implantable functional thick tissues require hierarchical vasculatures within cell‐laden hydrogel that can mechanically withstand the shear stress from perfusion and facilitate angiogenesis for nutrient transfer. Yet current extrusion‐based 3D printing strategies are unable to recapitulate hierarchical networks, highlighting the need for bioinks with tunable properties. Here, we introduce an approach whereby crosslinkable microgels enhance mechanical stability and induce spontaneous microvascular networks comprised of human umbilical cord vein endothelial cells (HUVECs) in a soft gelatin methacryoyl (GelMA)‐based bioink. Furthermore, we successfully implanted the 3D printed multi‐branched tissue, being connected from the rat carotid artery to the jugular vein direct surgical anastomosis. The work represents a significant step toward in the field of large vascularized tissue fabrication and may have implications for the treatment of organ failure in the future.

## Graphical abstract

The synergy of strength and biocompatibility in one matrix is essential to meet angiogenic HUVEC‐based sprouting and bear shear stress for implanting tissue engineering for clinical applications at a large scale. Here, the authors demonstrate a design strategy of microgel reinforced cell‐laden matrix with heterogeneous stiffness for constructing implantable vascular tissue using 3D bioprinting.

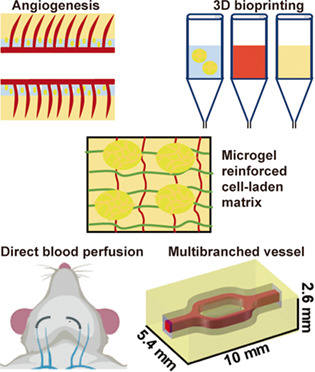

## 1 INTRODUCTION

Tissue engineering has shown tremendous potential for generating large functional tissue constructs as an alternative therapy for repairing or replacing damaged tissues and organs.^1^, ^2^, ^3^, ^4^, ^5^ Although advances in organoid technologies or organ‐on‐a‐chip have been made, the scale and complexity of bionic tissue still pose challenges that limit the applications.^6^, ^7^, ^8^, ^9^ In order to assure and promote the functions of the large engineered tissues, the fabrication of 3D vascular networks within the tissue constructs plays a critical role.

To date, only a few groups have attempted to bioprint the vessel‐like channels and capillary‐like structures in cell‐laden hydrogels.^10^, ^11^, ^12^ However, the mechanical strength of these hydrogels is typically insufficient to bear the shear stress of blood flow *in vivo*. While direct surgical anastomosis with immediate blood perfusion of vascularized tissues has been demonstrated using vascular stents or biodegradable scaffolds (e.g., POMAC), these approaches have limitations such as poor integration and potential biocompatibility issues.^13^, ^14^, ^15^

In 2021, our group proposed a multi‐material extrusion bioprinting method to print cell‐laden structures with an inner‐outer layer to achieve the immediate blood perfusion of vascularized tissues.^16^ Specifically, we utilized a 3GF (3% GelMA +0.25% fibrin) cell‐laden layer and a 5GM (5% GelMA) inner layer and outer layer structure to resist the shear stress of blood flow. However, the strength properties of 5GM were found not to be tough enough to support angiogenesis and hinder the delivery of nutrients from channel to tissue. This highlighted the need to synergistically address the demands of angiogenic sprouting *in vitro* and pressure‐bearing capacity *in vivo*.

**FIGURE 1:**
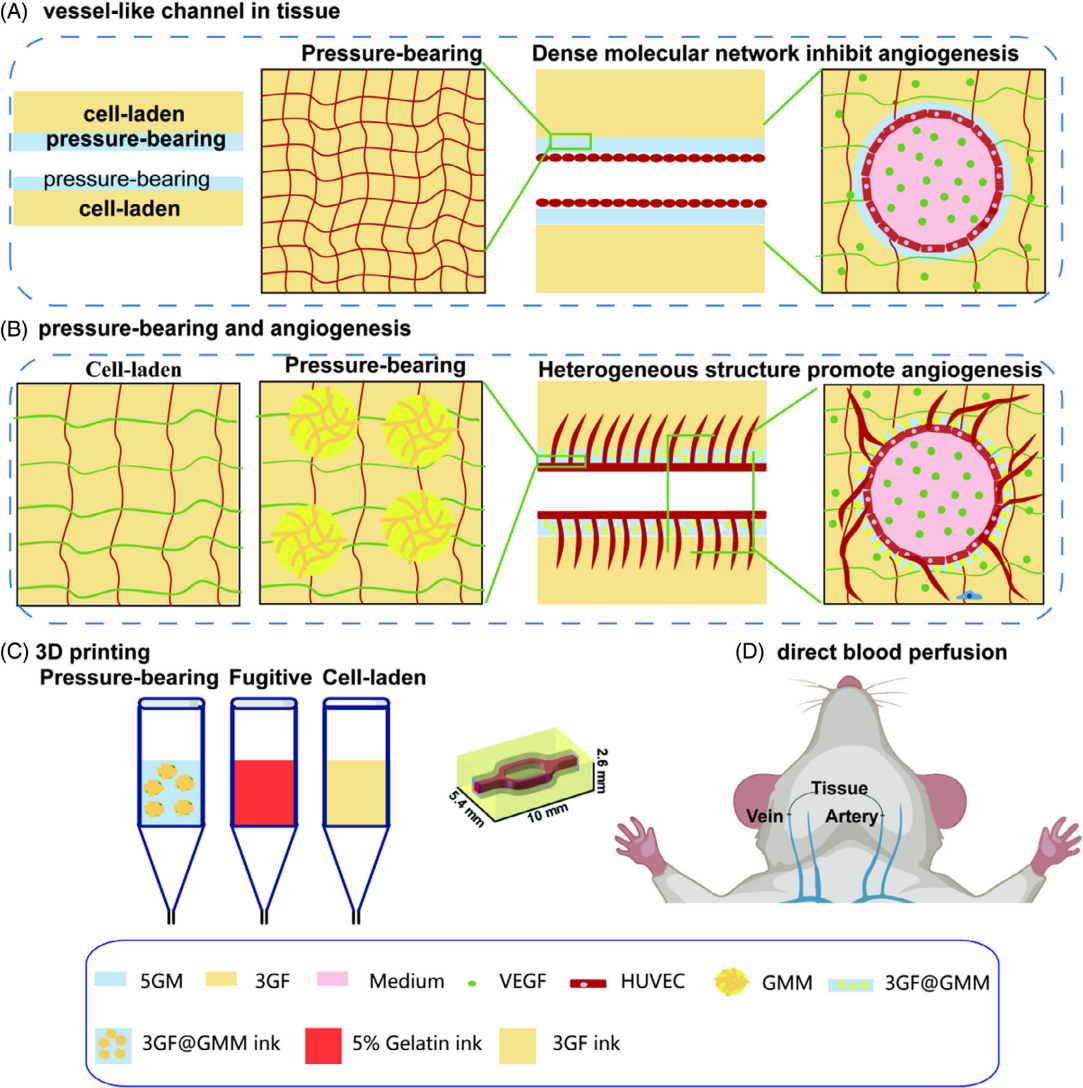
Schematic diagram of bioprintable cell‐laden hydrogels based on microgels for angiogenesis and pressure‐bearing ability. (A) Traditional pressure‐bearing hydrogels have dense crosslinking networks and thus inhibit endothelial angiogenesis. (B) Preliminary design of a pressure‐bearing cell‐laden hydrogel with chemically crosslinked GMM (yellow) enables angiogenesis. (C) Multi‐material bioprinting was performed using 3GF inks, fugitive inks, and 3GF@GMM inks to fabricate tissue with a multi‐branched vessel‐like channel. (D) A tissue scaffold was implanted *in vivo* by direct surgical anastomosis to the host vasculature (artery to vein).

Here, we present a novel strategy that employs hydrogel composites based on microparticles (Figure 1). By crosslinking GelMA microgels (GMM) with 3GF, we demonstrate that the resulting hydrogel composites exhibit spatially heterogeneous mechanical properties. Notably, the hydrogel composites enable angiogenic activities of HUVECs after endothelialization, essential for supplying nutrients from the channel to the 3D matrix. In addition, GMM improved the printability of 3GF, enhancing the versatility of the bioprinting process.

## 2 EXPERIMENTAL SECTION

### 2.1 Fabrication of GelMA microgels

GelMA was synthesized as previously described.^16^ Briefly, gelatin (type A, from porcine skin, G1890, Sigma‐Aldrich) was reacted with methacrylic anhydride (MA, Sigma‐Aldrich) in phosphate buffered saline (PBS). The resulting GelMA was used to prepare GelMA microgels (GMM) via a complex coacervation method. Specifically, 2 wt% of GelMA was dissolved in an ethanol aqueous solution (V (ethanol: H_2_O) = 17:8) and stirred with pluronic F127 (Sigma‐Aldrich) and gum arabic (Sigma‐Aldrich) added. at 0.125 and 0.025 times mass of GelMA, respectively. After adjusting pH to 6.2, the reaction was stirred at 300 rpm for 30 min at room temperature. The microgel‐containing solution was then centrifuged at 300 g for 5 min, and the microgels were washed with PBS and centrifuged at 3000 g for 10 min. The washing process was repeated three times before the microgels were stored at 4 °C for further use. To visualize the microgels, FITC‐NHS was used for modification. The mass concentration of GMM was determined by weighting the tube mass, the total mass after adding microgels, and the total mass after freeze‐drying.

### 2.2 Preparation of 3GF hydrogel composites with microparticles

To prepare 3GF hydrogel composites, GMM (0.4%) or phosphosilicate calcium bioactive glass (PSC, 0.5%) microparticles were mixed with 10% GelMA and fibrin at different ratios. Photo‐crosslinking was achieved by exposing the GelMA‐Fibrin, GelMA‐Fibrin + GMM, or GelMA‐Fibrin + PSC prepolymers to 10 mW cm^−2^ UV light (365 nm, Goodun) for 2 min.

### 2.3 Morphology and porosity analysis by scanning electron microscopy (SEM)

The morphology of 3GF, 5GM, 3GF + GMM, and 3GF + PSC were studied by SEM, respectively. Prior to imaging, the hydrogels were immersed in PBS at 37 °C for 12 h. Samples were then subjected to freeze‐fracturing by immersion in liquid nitrogen for 60 s, followed by sublimation at −75 °C for 90 min. Finally, samples were then sputter‐coated with gold and imaged with a scanning electron microscope (HITACHI S‐3000N&Quorum PP3000T). The pore areas of hydrogels were measured using the Analyse Particles function in ImageJ.

### 2.4 Mechanical properties testing

Nanoindentation experiments were performed using a nanoindenter (Piuma, Optics 11, Netherlands) to characterize Effective Young's modulus (Eeff) when hydrogels were immersed in PBS.

### 2.5 Cell culture and maintenance

Human umbilical cords were obtained from Xinhua Hospital affiliated with Shanghai Jiao Tong University School of Medicine after obtaining informed consent from all participants and approval from the institutional ethical committee (approval number: XHEC‐C‐2020‐092‐1). Human umbilical cord mesenchymal stem cells (MSCs) were donated by the National Stem Cell Resource Centre, Beijing. The transduction method with red fluorescent protein (RFP) into HUVECs were as previously reported.^17^ RFP‐expressing HUVECs (RFP‐HUVECs) were cultured in EMG2 medium (complete EGM‐2 BulletKit™, Lonza). Green fluorescent protein‐expressing MSCs (GFP‐MSCs) were cultured in Dulbecco's modified Eagle medium containing high glucose and sodium pyruvate (DMEM, Gibco) supplemented with 15% fetal bovine serum (FBS, Bioind), 1% non‐essential amino acid solution (NEAA, Gibco), 1% GlutaMAX™ (Gibco), and 1% penicillin/streptomycin (Gibco). The culture medium was added and changed every 2 days for culturing at 37 °C with 5% CO_2_. RFP‐HUVECs and GFP‐MSCs were not used beyond the 10th passage.

### 2.6 Biocompatibility study by encapsulation of MSCs in hydrogels

To test the biocompatibility of 3GF@GMM and 3GF@PSC, 1 × 10^6^ mL^−1^ GFP‐MSCs were collected and mixed with 3GF, 3GF@GMM, and 3GF@PSC prepolymers, respectively. The cell‐prepolymer mixture (20 μL) was dispensed in each well of a 24‐well flat bottom cell culture plate and photo‐crosslinked by exposing to 10 mW cm^−2^ UV light (365 nm, Goodun) for 2 min. The encapsulated hydrogels were then cultured with EGM‐2 medium for 48 h, after which a fluorescence microscope imaged the morphology of cells in hydrogels. Finally, the cells were stained for 30 min with Texas red‐phalloidin imaged using a laser confocal microscope after being processed with with 4% paraformaldehyde and 0.5% Triton‐100 for 30 min, respectively.

### 2.7 Endothelial monolayer and angiogenesis studies

All hydrogel pre‐polymers were supemented with 40 ng mL^−1^ basic fibroblast growth factor (bFGF, R&D Systems) and 40 ng mL^−1^ vascular endothelial growth factor (VEGF, R&D Systems) before cross‐linking. The angiogenesis assay was performed using 3D printed vascular pipeline moulds. First, an 800‐μm‐diameter needle was inserted into the mould and reversed, and hydrogel composite prepolymers were added and cross‐linked using UV light to create the vascular‐like microchannel with an 800 μm diameter. RFP‐HUVECs with a density of 4 × 10^6^ cells mL^−1^ in 10 μL medium with 10 μg mL^−1^ fibronectin were added into the microchannels and allowed to adhere to the microchannel surface for 10 min. The process was repeated four times for different surfaces of the microchannel to ensure complete coverage. Unattached cells were removed by washing with fresh media. The mould was then placed on a plate rocker (BenchRocker BR2000) at 10 rpm for 24 h. The medium was replaced with EGM2 with 40 ng mL^−1^ VEGF and 40 ng mL^−1^ bFGF and incubated for several days for the angiogenic sprouting assay before further characterization. Cell culture media and growth factor cocktails were replenished twice daily.

### 2.8 Rheological measurements

The rheological behaviour of 3GF hydrogels with or without GMM was characterized using oscillatory shear rheometry with parallel plate geometry (Anton Paar, MCR 302).^18^ Flow sweeps were performed at shear rates ranging from 0.01 to 100 s^−1^ to determine apparent viscosities. Temperature sweep (oscillation) was used to determine the temperature dependence of storage (G′) and loss modulus (G′′) by increasing the temperature from 4 to 25 °C at a rate of 5 °C min^−1^.

### 2.9 3D printing vascular channel structure in large volume tissue

The printing method was performed as previously described.^16^ For transplantation printing, 5% gelatin, 3GF, and 3GF + GMM were used as sacrificial, cell‐laden, and elastic materials, respectively. 3GF + GMM was used as inner elastic inks and external elastic inks outside fugitive inks and cell‐laden inks, respectively.

### 2.10 Implantable property experiment

The ability of 3GF@GMM to bear the shear stress from blood flow and the implantability of the tissue was demonstrated using an artery‐to‐vein configuration. The tissue was encapsulated in PDMS and successfully implanted *in vivo* by direct surgical anastomosis.

### 2.11 Statistical analysis

All data were expressed as mean ± standard deviation (SD). The statistical analysis was performed using Origin or GraphPad Prism. All figures were obtained from three independent experiments with similar results.

## 3 RESULTS AND DISCUSSION

### 3.1 Fabrication and characterization of 3GF@GMM and 3GF@PSC hydrogels

**FIGURE 2:**
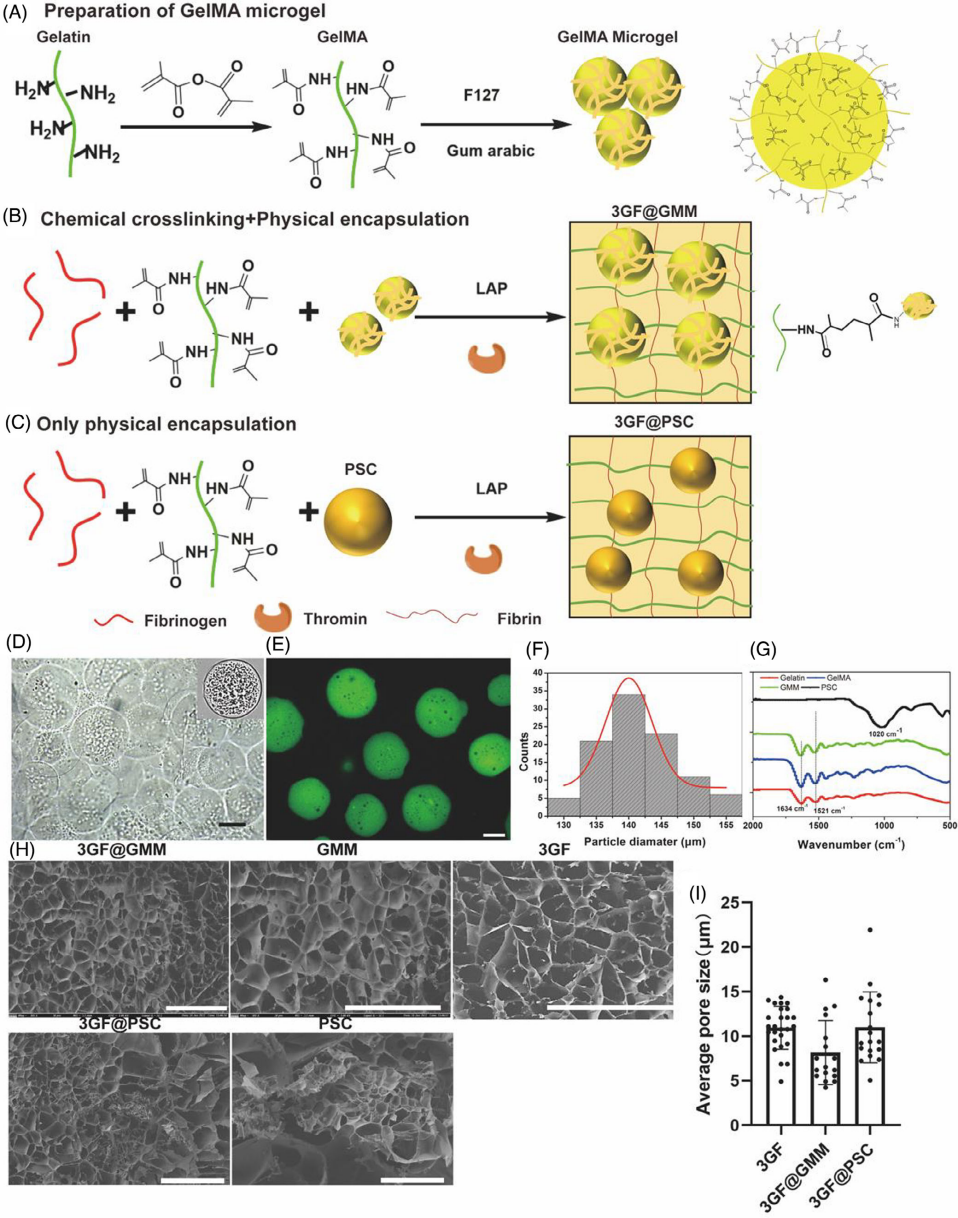
Characterization of 3GF@GMM and 3GF@PSC. (A) Schematic diagram depicting steps involved in preparation of GMM. (B) Fabrication of 3GF@GMM by GMM chemically crosslinked with GelMA in 3GF after physical encapsulation. (C) Fabrication of 3GF@PSC by pre‐physically wrapping phosphosilicate calcium bioactive glass (PSC) in 3GF before crosslinking. (D) Optical image of GMM. (Scale bar: 100 μm) (E) Representative confocal fluorescence image of GMM after modification with FITC‐NHS. (Scale bar: 100 μm) (F) Statistical distribution analysis of GMM diameter. (G) FI‐IR results of GelMA, GMM, and PSC (H) SEM images of 3GF@GMM, GMM, 3GF, PSC (Scale bar: 10 μm), 3GF@PSC and magnification image of PSC in 3GF. (Scale bar: 100 μm) (I) Quantitative analysis of the average pore size in the hydrogel.

GMMs with uniform morphologies were synthesized through a complex coacervation method using 70% methacrylation of gelatin (^1^H NMR data has been shown in our previous work^16^) (Figure 2A). After modification with FITC‐NHS, the spherical GMM exhibited a uniform morphology with an average diameter of 140 μm (Figure 2D,F). The spherical structure was more suitable for forming heterogeneity after crosslinking with GelMA chains (Figure 2E). The morphologies of GelMA and GMM powder after freeze‐drying were also displayed in Figure S1.

While 3GF hydrogel has soft mechanical properties that could not bear physiological fluid shear stress, the addition of 5% GelMA improve the pressure‐bearing properties of 5GF, but reduced bioactivity due to densely crosslinked networks (Figure S2). To enhance mechanical properties of 3GF while maintaining cell‐laden properties, GMM were added to 3GF, which involved physical encapsulation and chemical crosslinking between GMM and GelMA, and using 0.1% w/v lithium phenyl‐2,4,6‐trimethylbenzoylphosphinate (LAP) as a photo‐initiator, and fibrinogen polymerization into fibrin with thrombin (Figures 2B and S3). SEM results showed that 3GF@GMM exhibited evident heterogeneous structure, with GMM displaying a densely crosslinked among the loosely crosslinked structure of 3GF (Figure 2H). The microgels were stable in the whole gel structure due to the GMM chemical crosslinking between GMM and GelMA chains.

In addition, phosphosilicate calcium bioactive glass (PSC) were added to 3GF mixture to form 3GF@PSC by simple physical encapsulation (Figure 2C).^19^ The configuration of GelMA, GMM, and PSC was characterized using fourier transform infrared (FT‐IR) spectroscopy. After MA modification, the resulting GelMA and GMM exhibited a C=O stretching vibration band at 1634 cm^−1^ (amide I band) and N‐H stretching vibration and bending vibration band at 1521 cm^−1^ (amide II band) than gelatin (Figure 2G).^20^ PSC exhibited a typical Si‐O‐Si stretching vibration band at 1020 cm^−1^. The SEM images revealed that the average size of interconnected pores decreased after the addition of GMM or PSC, with the size distribution of pores exhibiting heterogeneity due to GMM internal crosslinking and GMM‐GelMA crosslinking (Figure 2H,I).

### 3.2 Stiffness and biocompatibility of 3GF@GMM and 3GF@PSC

**FIGURE 3:**
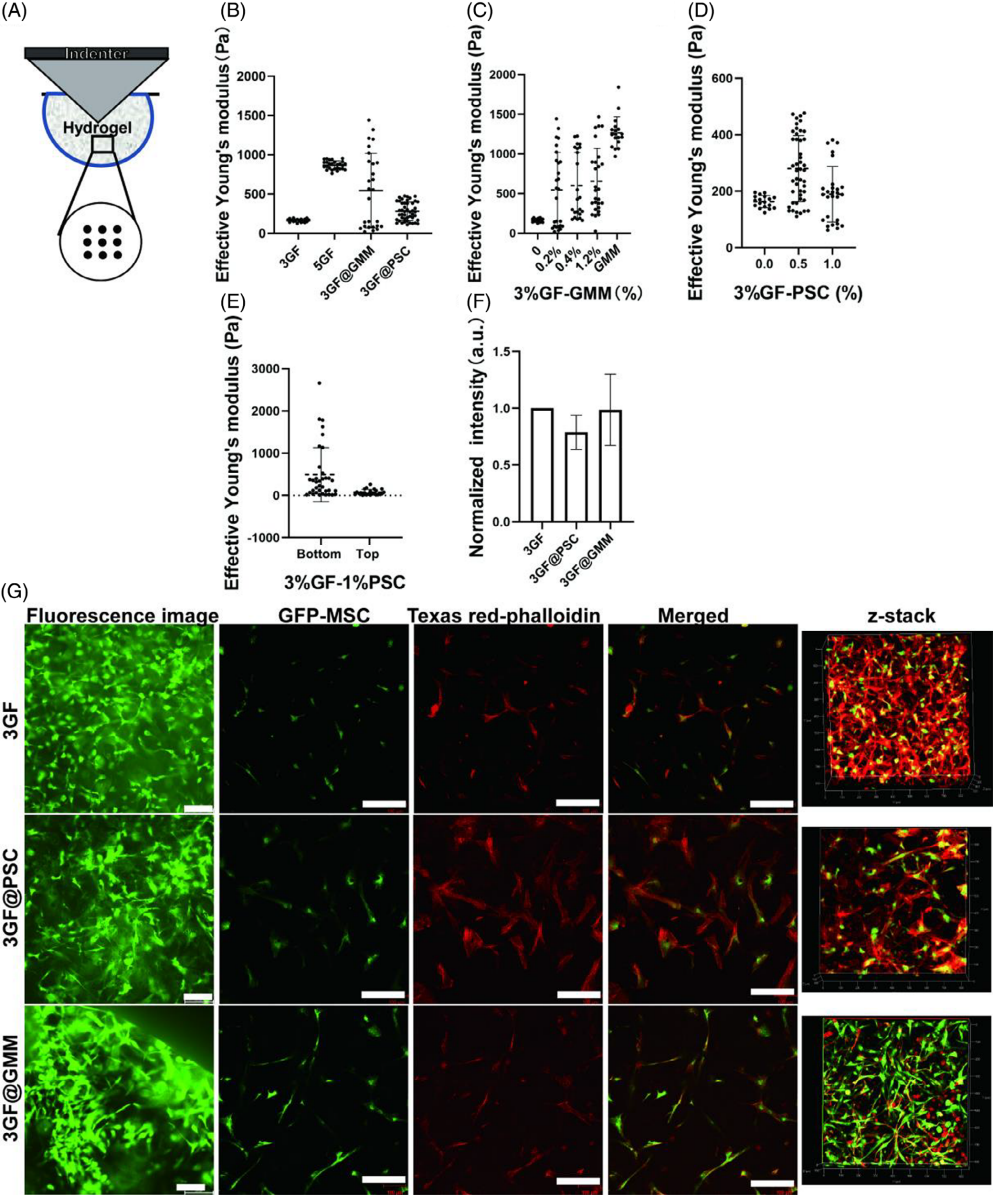
Characterization of stiffness and biocompatibility of 3G, 3GF@GMM, and 3GF@PSC hydrogels. (A) Schematic representation of the Eeff testing. (B) Eeff of 3GF, 5GF, 3GF@GMM, and 3GF@PSC hydrogels. (C) Eeff of 3GF with different concentrations of GMM (0, 0.2%, 0.4%, 1, 2%) and pure 4% GMM hydrogels. (D) Eeff of 3GF at various PSC concentrations (0, 0.5%, 1%). (E) Eeff of top and bottom surfaces of 3%GF‐1%PSC. (F) Normalized fluorescence intensity of GFP‐MSCs in 3GF, 3GF@PSC, and 3GF@GMM for 48 h cultures. (*n* = 3, mean ± SD) (G) Fluorescence images of GFP‐MSCs in 3GF, 3GF@PSC, and 3GF@GMM for 48 h culture (left), respectively. GFP‐MSC confocal fluorescence images (green), MSC Actin cytoskeleton stained with Texas red‐phalloidin (red), and MSC 3D‐stacked images. (Scale bar: 200 μm).

The mechanical properties of 3GF@GMM and 3GF@PSC were studied through stiffness test using a nanoindentation device (Piuma Inc., Optics 11), 3GF and 5GF were used as a positive and negative control, respectively.^21^ The testing process was shown schematically in Figure 3A. The material testing revealed the increased mean E‐modulus after the addition of GMM or PSC (3GF: 163 ± 21.4 Pa, 5GF: 867.7 ± 50.8 Pa, 3GF@GMM: 544.7 ± 473.8 Pa, and 3GF@PSC: 280.1 ± 116.8 Pa, Figure 3B). Compared with the pure GMM and 3GF hydrogels, the Eeff of 3GF@GMM presented an evident multi‐point distribution, with the concentration of GMM increasing in 3GF, which could be due to the structural heterogeneous (Figure 3C). However, when the concentration of PSC increased, the stiffness of the bottom and top surfaces of 3GF@PSC varied (Figure 3D,E). The difference may be attributed to the enhanced precipitating efficiency of PSC microparticles in 3GF during the crosslinking process. So, we chose 0.5% PSC in 3GF for further use. Therefore, the physical encapsulation and chemical crosslinking between GMM and 3GF presented a more stable heterogeneous structure and higher average stiffness than pure physical enclosure PSC in 3GF.

It is widely known that high crosslinking density favours dense structure and enhanced stiffness.^22^, ^23^, ^24^ Hydrogel stiffness has also been demonstrated to affect cell activities and functions, such as cellular morphology,^25^ proliferation,^26^ migration,^27^ differentiation,^28^ stemness,^29^ and so forth. To study biocompatibility, GFP‐MSCs were encapsulated in 3GF, 3GF@GMM, and 3GF@PSC, respectively. Fluorescence images and normalized fluorescence intensity both showed MSCs spreading and extending excellently in all groups (Figures 3F,G and S4). This phenomenon was confirmed by confocal microscopy images, in which the cytoskeleton was clearly observed in the 3D hydrogel matrix and the fibroblast‐like morphology of MSCs. In addition, RFP‐HUVECs could self‐assemble when cultured in the 3GF@GMM matrix using HUVEC‐MSC aggregates (Figure S5).

### 3.3 Endothelialization and angiogenesis in the 3GF@GMM and 3GF@PSC constructed vessel‐like channels

**FIGURE 4:**
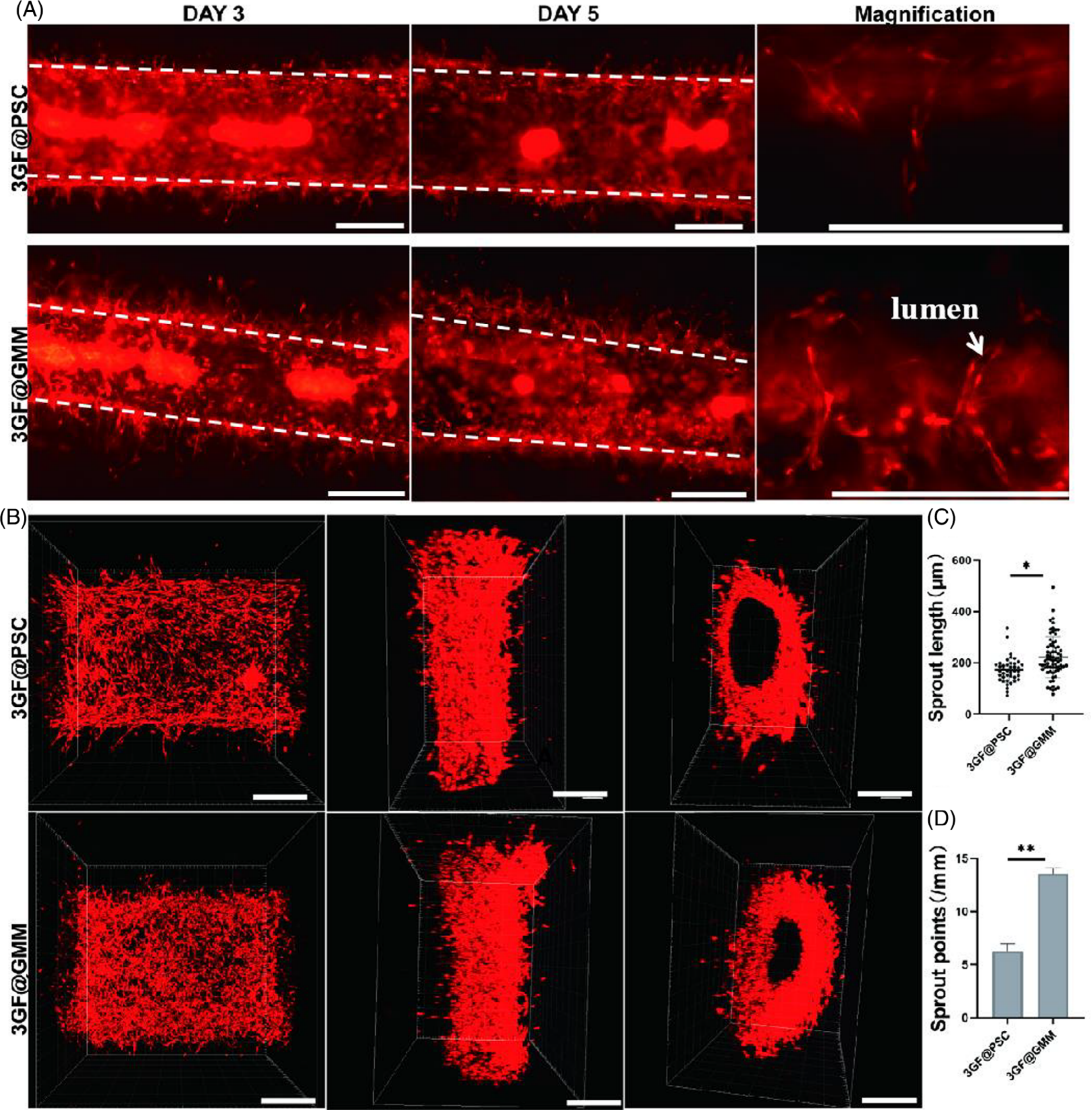
Characterization of endothelialized micronetworks and angiogenesis of 3GF@PSC and 3GF@GMM. (A) Top view of fluorescence images of RFP‐HUVECs spreading and sprouting on an 800‐μm‐diameter microchannel for 3 and 5 days of culture, respectively (left). Side view magnification (right) (B) and representative 3D constructed confocal fluorescence image of RFP‐HUVECs on microchannel for 5 days of culture (top view, side view, and inter microchannel view) Quantification of HUVECs sprout length (C) and sprout points after 5 days of culture (D). (Scale bar: 500 μm).

It is meaningful for tissue engineering to develop hydrogels that support angiogenesis for supplying nutrients and oxygen from host vasculature to thick tissue of nutrients and oxygen.^30^ Natural and synthetic materials with low stiffness have been studied to support angiogenesis.^31^, ^32^, ^33^, ^34^ Previously, we showed that the 3GF matrix supported angiogenic HUVEC‐based invasions.^16^ While 3GF hydrogel has soft mechanical properties, encapsulation of GMM or PSC in 3GF matrix increases matrix stiffness and may affect cell migration and angiogenesis. The sprouting of angiogenesis from a HUVECs‐coated microchannel was studied. No appreciable invasion occurred in 5GF hydrogels, while 3GF@PSC and 3GF@GMM matrixes both stimulated HUVECs to invade hydrogels (Figure 4A, S6). Sprout length increased as culture time increased. From the magnification images, we could see the sprouting structures that recapitulate angiogenesis, forming lumens lined by multiple cells. Furthermore, when the PSC concentration in the 3GF@PSC matrix was increased to 1%, the sprouting phenomenon patterns on the upper and lower surfaces of the same channel were clearly different. The results were consistent with hydrogel stiffness variation (Figures 3E and S6). Quantification of sprouting length and density (points mm^−1^) illustrated that 3GF@GMM matrix showed better supporting angiogenic sprouting properties than 3GF@PSC, which may be attributed to the stable heterogeneous stiffness and porous distribution (Figure 4C,D). 3D‐reconstructed confocal microscopy images confirmed the presence of endothelial attachment, tight cell–cell contacts on the channel surface, and cells invading the surrounding matrix after 5 days of culture (Figures 4B and S7).

### 3.4 3D printing of vascular constructs and implantation *in vivo*

**FIGURE 5:**
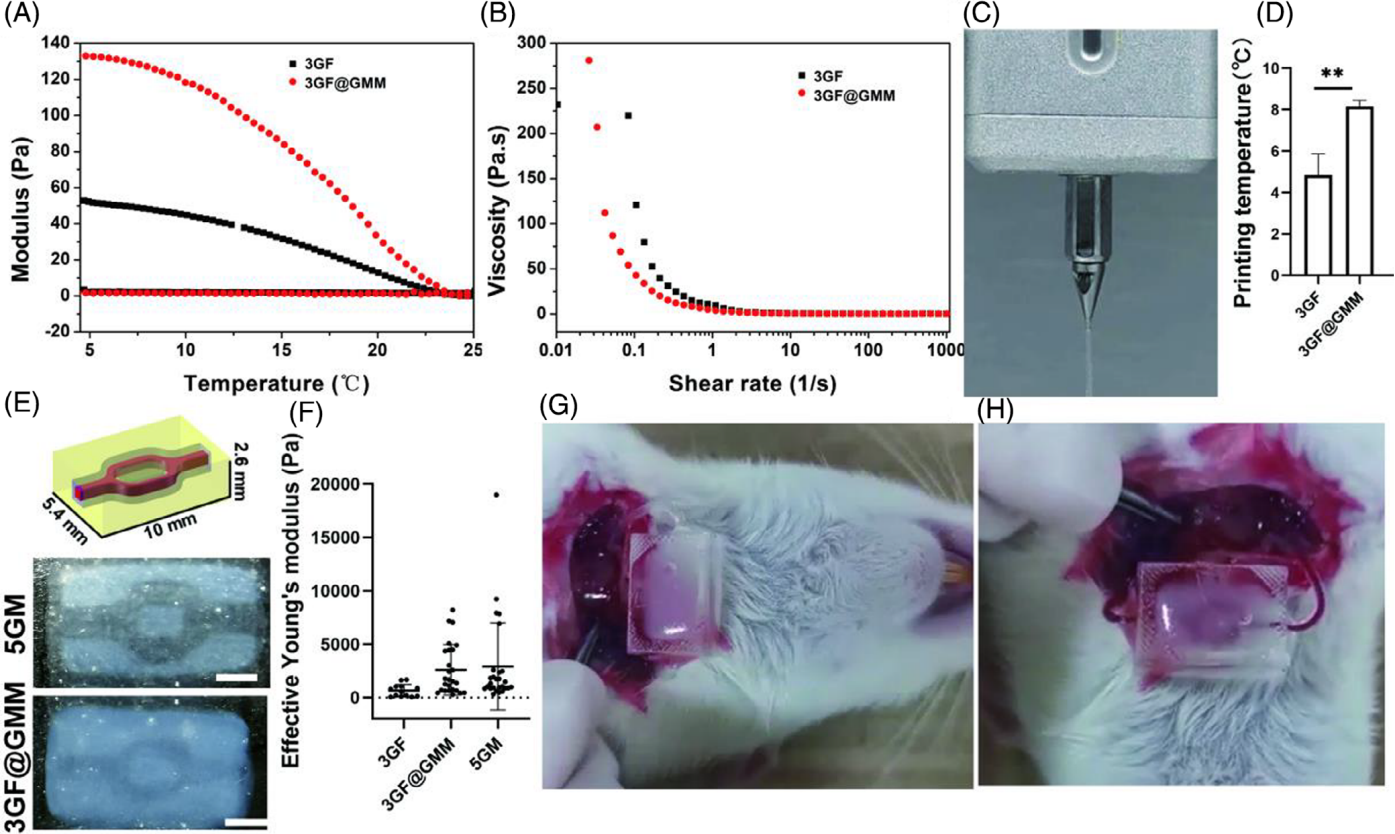
3D printing of vascular constructs *in vitro* and implantation *in vivo*. G′ and G″ values as functions of temperature (A) and viscosity as a function of shear rate (4 °C) (B) of the 3GF@GMM and 3GF bioinks. (C) 3GF@GMM printable phase at 8 °C. (D) Statistical analysis of printing temperatures for 3GF@GMM and 3GF bioink (*n* = 3, mean ± SD, *p < 0.1, **p < 0.01, ***p < 0.001). (E) Printing model: 5GM and 3GF@GMM as inner layers for constructing thick tissue with the ‘one‐to‐two’ channel, respectively. (F) Eeff of the printed constructs with 3GF, 3GF@GMM, and 5GM as elastic layers, respectively. Observation of implanted tissues after establishing anastomosis with the carotid artery and jugular vein (G) and direct blood perfusion after implant (H).

In addition, the effect of GMM on the printing properties of 3GF@GMM was studied through rheological measurements. Certain concentrations of GelMA must be used to achieve appropriate printability. GMM could complement GelMA when combined, acting as a viscosity and storage modulus enhancer to improve printability (Figure 5A,B). We built on a previously established BiopHead printing system for constructing thick tissue (10 × 5.4 × 2.6 mm) with the ‘one‐to‐two’ channel using elastic inks (5GM), cell laden inks (3GF), and fugitive inks (5% gelatin). After implantation, the elastic layer serves as the inner layer to bear shear stress from physiological blood flow for direct surgical anastomosis to host vasculature *in vivo*. Because 5GM could not support angiogenic HUVECs‐based sprouting to limit nutrient diffusion, 3GF@GMM were used as elastic inks in this study instead of 5GM, and the temperature control system was adjusted to keep the bioink in the gel state (Figure 5C). According to the printing temperature, 3GF@GMM exhibited lower requirements for printing temperature than 3GF system (3GF: 4.8 ± 1.0 °C, 3GF@GMM: 8.2 ± 0.3 °C, Figure 5D). The printing tissue constructs that used 3GF@GMM as elastic layer exhibited nearly consistent with tissue printed with 5GM as elastic layer (Figure 5E). Finally, to assure the pressure‐bearing ability after implantation by direct surgical anastomosis, we further evaluated the stiffness of printed tissues by testing their Eeff. The Eeff of tissue using 3GF@GMM as the elastic layer is similar to that of tissue using 5GM as elastic layer (Figure 5F). Before being implanted *in vivo*, the blood compatibility of 3GF@GMM, which comes into direct contact with the blood surface, was also investigated (Figure S8). In artery‐to‐vein mode, the printed constructs encapsulated in poly(dimethylsiloxane) (PDMS) were successfully connected to the arteria vessel of adult Sprague–Dawley (SD) rats (Figure 5G). After that, the implanted tissue was stable after direct blood perfusion, which indicated that the 3GF@GMM could bear the shear stress from physiological blood flow (Figure 5H, Movie S1). Specifically, the anastomosis of prevascularization tissue constructs to the host vasculature with perfusion typically takes several days after implantation, as reported in previous studies.^17^, ^35^, ^36^, ^37^ However, these methods alone cannot prevent ischemic cell death within larger 3‐dimensional tissue substitutes during the initial days following implantation. In contrast, our bioprinted tissue can be directly perfused with blood by surgical anastomosis to the host vasculature. Compared to previous reports, our approach represents a significant advancement in addressing the issue of ischemic cell death in larger tissue substitutes. Above all, the 3GF@GMM matrix could support angiogenesis *in vitro* and withstand blood fluid shear stress *in vivo*, which is critical to the constructed large tissue with the *in vitro* built vascular network for direct nutrient supply from blood *in vivo*.

## 4 CONCLUSIONS

We have successfully developed a strategy by incorporating micrometre‐scale hydrogel microspheres (GMM) crosslinked within cell‐laden matrix (3GF) to enhance angiogenesis *in vitro* and bear shear stress of physiological blood flow *in vivo*. Compared to physical encapsulation of PSC in 3GF, GMM chemical crosslinking in 3GF obviously increased the stiffness and built a more heterogeneous microenvironment with tunable porosity and stiffness, affecting MSC spreading and HUVECs‐based sprouting. GMM also improved the viscosity and storage modulus of 3GF, enhancing its printability. The printed constructs with vascular networks using 3GF@GMM as a pressure‐bearing layer were successfully implanted and perfused with blood *in vivo*, demonstrating the ability of 3GF@GMM to withstand shear stress from blood flow. Our approach provides a promising strategy for constructing thick, implantable, functional tissue with multi‐branched vascular networks using cell‐laden hydrogels reinforced with microgels via physical and chemical crosslinking strategies.

## AUTHOR CONTRIBUTIONS

Xinhuan Wang and Xin Liu, contributed equally to this work. Ailing Li and Dong Qiu provided PSC. Qi Gu conceived and designed the project together with Xiongfei Zheng, Xinhuan Wang and Xin Liu; Xinhuan Wang, Xin Liu, Wenli Liu and Yanyan Liu performed experiments. Xinhuan Wang, Xin Liu, and Qi Gu collected, analysed the data and wrote the paper.

## CONFLICT OF INTEREST STATEMENT

The authors declare no conflict of interest.

## Supporting information


**Figure S1.** SEM images of GelMA (A) and GMM (B) after freeze‐drying, respectively. (Scale bar: 20 μm)
**Figure S2.** SEM image of 5GF hydrogel (5%GelMA+0.25%Fibrin). (Scale bar: 100 μm)
**Figure S3.** The observation of GMM in 3GF@GMM hydrogel under the light microscope.
**Figure S4.** The observation of GFP‐MSCs morphology in 3GF, 3GF@PSC, and 3GF@GMM hydrogels cultured for 7 days under the fluorescence microscope, respectively. (Scale bar: 200 μm)
**Figure S5.** The observation of RFP‐HUVECs after coculuted with MSCs in 3GF@GMM hydrogel for 3 days. Confocal image (left) and SEM images (right).
**Figure S6.** Characterization of endothelialized micro‐networks and angiogenesis of 3GF@PSC and 5GF with 40 ng mL^−1^ VEGF and 40 ng mL^−1^ bFGF. (Scale bar: 200 μm)
**Figure S7.** SEM images of HUVECs attached on the surface of channels. (Scale bar: 20 μm)
**Figure S8.** Blood‐compatibility study of 3GF@GMM. (A)Hemolysis rate. (B) Recalcification time. The microscope image of 3GF@GMM in printed constructs as a pressure‐bearing layer. (*n* = 3, mean ± SD, *p < 0.1, **p < 0.01, ***p < 0.001)

**Movie S1:** Observation of transplanted tissues after the establishment of connections with carotid artery and jugular vein showing arterial clip slip and establishment of blood perfusion.

## Data Availability

The data that support the findings of this study are available from the corresponding author upon reasonable request.
