# Supplementary material for: 3D bioprinting microgels to construct implantable vascular tissue
Source: Cell Prolif. 2023 May 17;56(5):e13456. doi: 10.1111/cpr.13456 (PMC10212694; doi:10.1111/cpr.13456)
Supplement: Supplementary file 1 — Figure S1. SEM images of GelMA (A) and GMM (B) after freeze‐drying, respectively. (Scale bar: 20 μm) Figure S2. SEM image of 5GF hydrogel (5%GelMA+0.25%Fibrin). (Scale bar: 100 μm) Figure S3. The observation of GMM in 3GF@GMM hydrogel under the light microscope. Figure S4. The observation of GFP‐MSCs morphology in 3GF, 3GF@PSC, and 3GF@GMM hydrogels cultured for 7 days under the fluorescence microscope, respectively. (Scale bar: 200 μm) Figure S5. The observation of RFP‐HUVECs after coculuted with MSCs in 3GF@GMM hydrogel for 3 days. Confocal image (left) and SEM images (right). Figure S6. Characterization of endothelialized micro‐networks and angiogenesis of 3GF@PSC and 5GF with 40 ng mL−1 VEGF and 40 ng mL−1 bFGF. (Scale bar: 200 μm) Figure S7. SEM images of HUVECs attached on the surface of channels. (Scale bar: 20 μm) Figure S8. Blood‐compatibility study of 3GF@GMM. (A)Hemolysis rate. (B) Recalcification time. The microscope image of 3GF@GMM in printed constructs as a pressure‐bearing layer. (n = 3, mean ± SD, *p < 0.1, **p < 0.01, ***p < 0.001) [file CPR-56-e13456-s002.docx]

SUPPORTING INFORMATION

**3D Bioprinting microgels to construct implantable vascular tissue**

Xinhuan Wang,^[-]a^ Xin Liu,^[-]a^ Wenli Liu,^a^ Yanyan Liu,^a,b^ Ailing Li,^c^ Dong Qiu,^c,d^ Xiongfei Zheng*,^d,e^ Qi Gu*^a,d,f^

a. State Key Laboratory of Membrane Biology, Institute of Zoology, Chinese Academy of Sciences, Chaoyang District, Beijing 100101, P. R. China;

b. School of Materials Design and Engineering, Beijing Institute of Fashion Technology, Chaoyang District, Beijing 100029, P. R. China

c. Beijing National Laboratory for Molecular Sciences, State Key Laboratory of Polymer Physics and Chemistry, Institute of Chemistry, Chinese Academy of Sciences, Haidian District, Beijing 100190, P. R. China

d. University of Chinese Academy of Sciences, Huairou District, Beijing 101449, P. R. China

e. Shenyang Institute of Automation, Chinese Academy of Sciences, Hunnan District, Shenyang 110169, P. R. China

f. Beijing Institute for Stem Cell and Regenerative Medicine, Chaoyang District, Beijing 100101, P. R. China.

* Corresponding Email: [qgu@ioz.ac.cn](mailto:yangr@nanoctr.cn,%20wangch@nanoctr.cn) and zhengxiongfei@sia.cn.

† Supporting information for this article is available

[-] These authors contributed equally to this work.


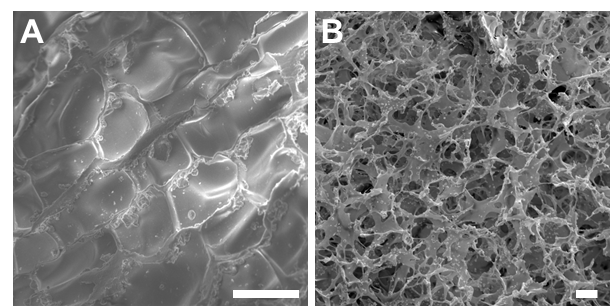


Figure S1. SEM images of GelMA (A) and GMM (B) after freeze-drying, respectively. (Scale bar: 20 μm)


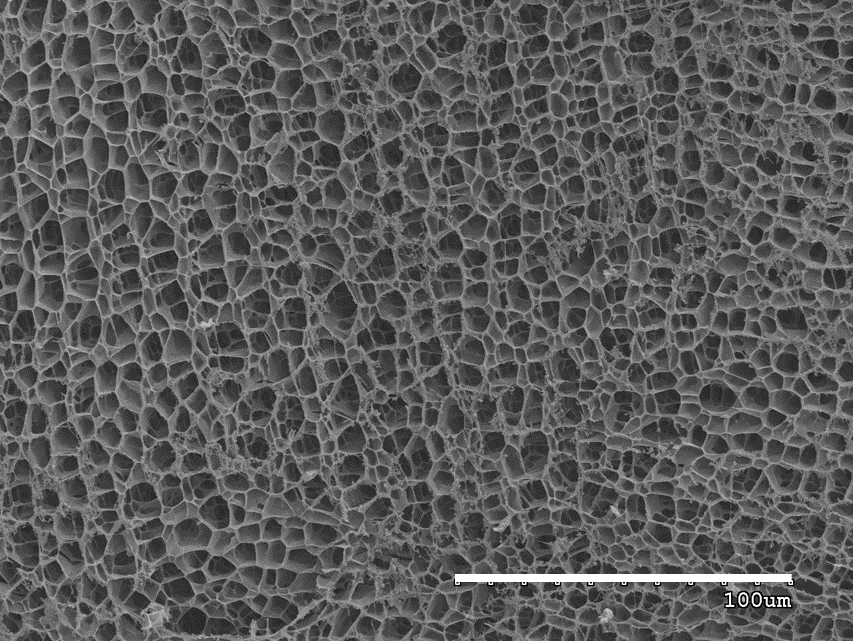


Figure S2. SEM image of 5GF hydrogel (5%GelMA+0.25%Fibrin). (Scale bar: 100 μm)


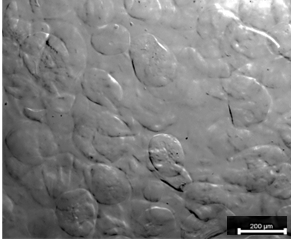


Figure S3. The observation of GMM in 3GF@GMM hydrogel under the light microscope.


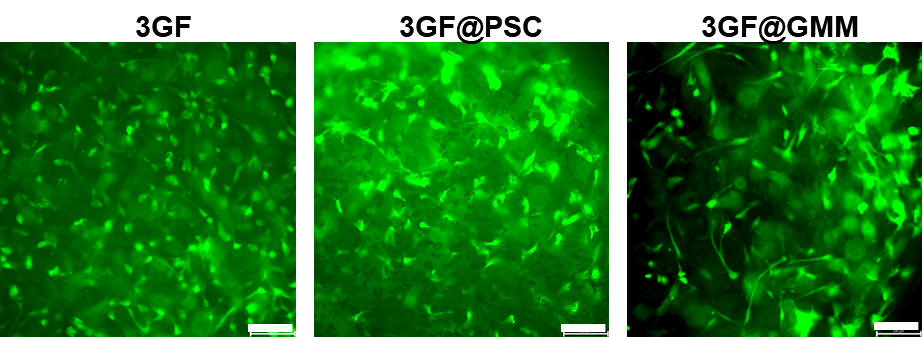


Figure S4. The observation of GFP-MSCs morphology in 3GF, 3GF@PSC, and 3GF@GMM hydrogels cultured for 7 days under the fluorescence microscope, respectively. (Scale bar: 200 μm)


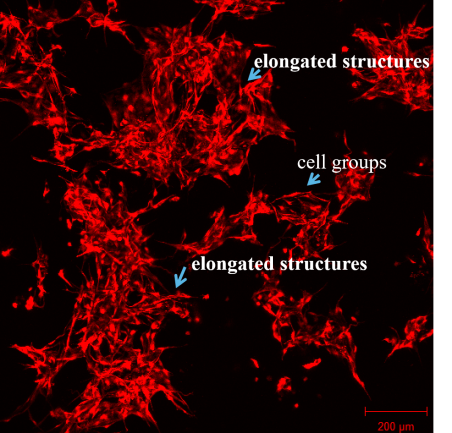

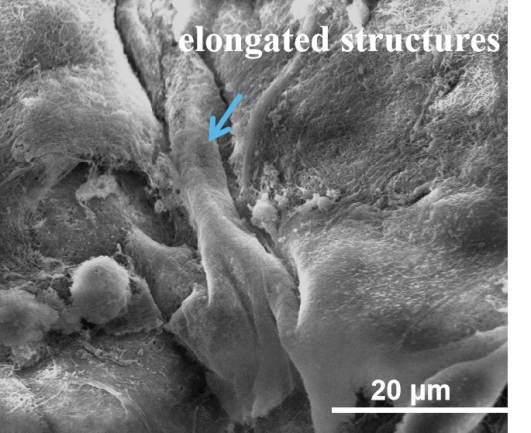


Figure S5. The observation of RFP-HUVECs after coculuted with MSCs in 3GF@GMM hydrogel for 3 days.Confocal image (left) and SEM images (right).


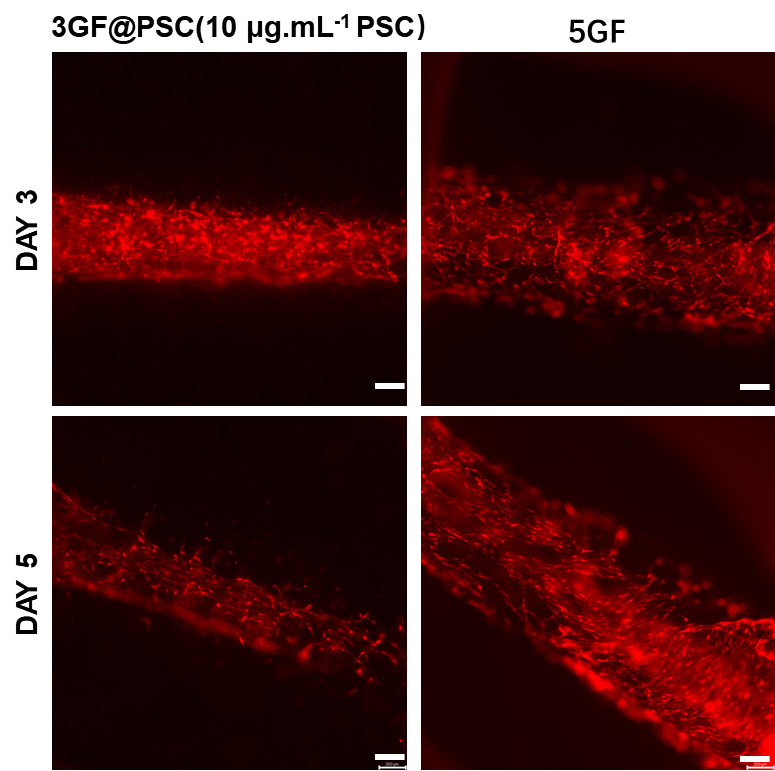


Figure S6. Characterization of endothelialized micro-networks and angiogenesis of 3GF@PSC and 5GF with 40 ng mL^−1^ VEGF and 40 ng mL^−1^ bFGF. (Scale bar: 200 μm)


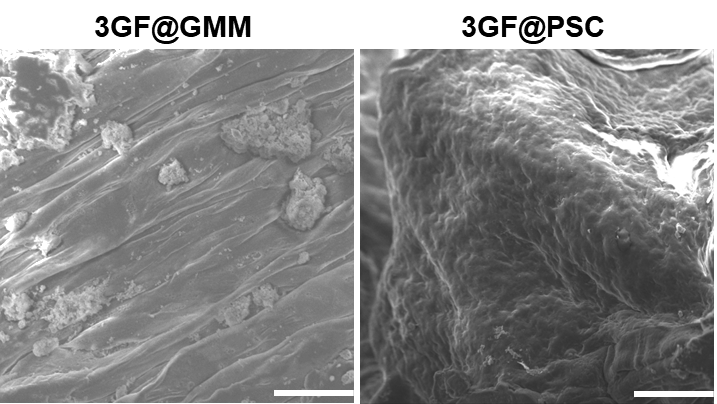


Figure S7. SEM images of HUVECs attached on the surface of channels. (Scale bar: 20 μm)


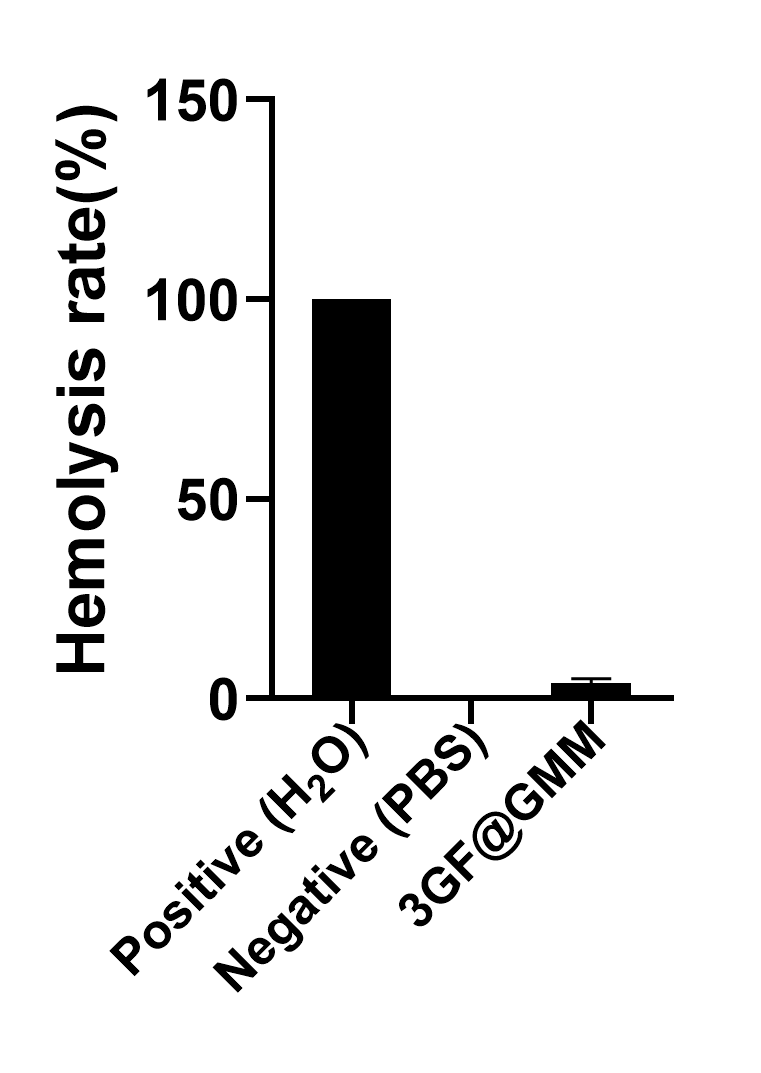

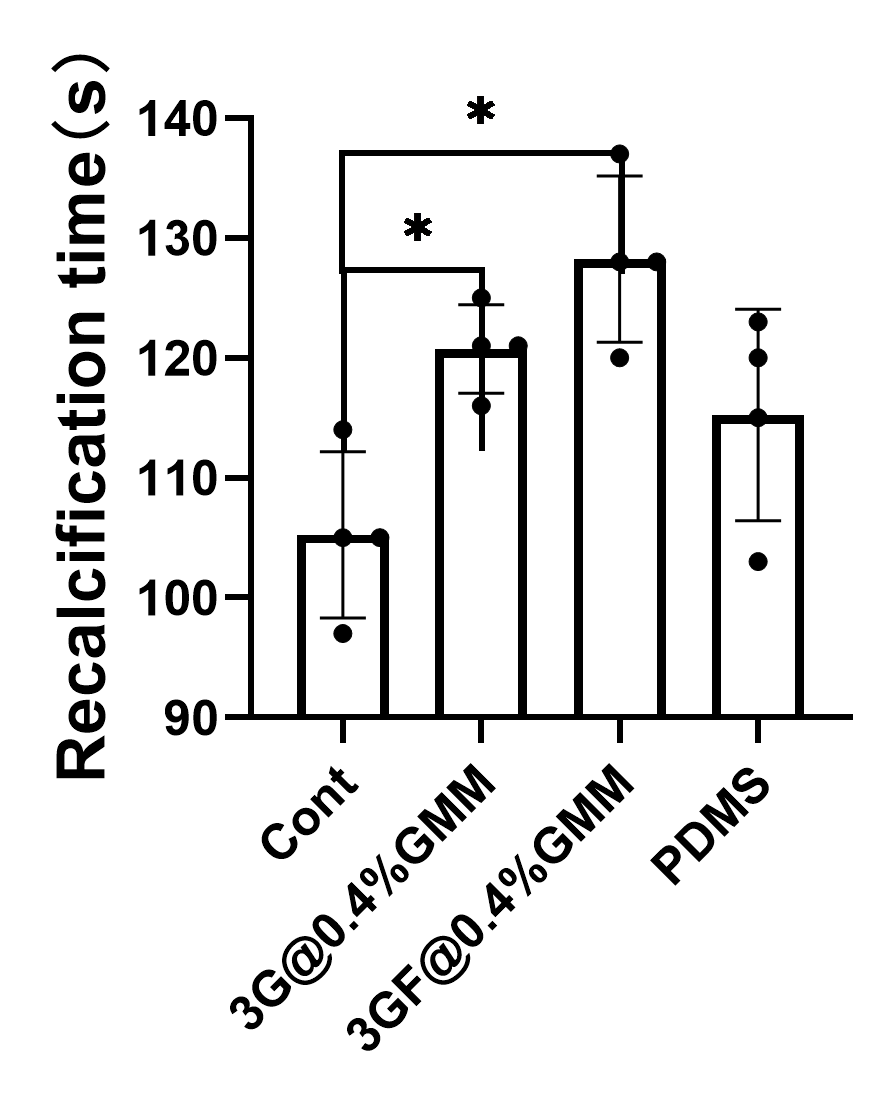


Figure S8. Blood-compatibility study of 3GF@GMM. (A)Hemolysis rate. (B) Recalcification time. The microscope image of 3GF@GMM in printed constructs as a pressure-bearing layer. (n = 3, mean ± SD, * *p* < 0.1, ** *p* < 0.01, *** *p* < 0.001)

**Supplementary movies captions:**

**Movie S1:** Observation of transplanted tissues after the establishment of connections with carotid artery and jugular vein showing arterial clip slip and establishment of blood perfusion.
